# Antibiotic resistance in Kurdistan, Iraq: A growing concern

**DOI:** 10.1016/j.nmni.2024.101221

**Published:** 2024-01-24

**Authors:** Karzan Qurbani, Seenaa Ali, Safin Hussein, Haider Hamzah

**Affiliations:** Department of Biology, College of Science, University of Raparin, Rania, Sulaymaniyah, 46012, Iraq; Department of Medical Laboratory, College of Health and Medical Technologies, Sulaimani Polytechnic University, Sulaymaniyah, Kurdistan Region, Iraq; Department of Biology, College of Science, University of Raparin, Rania, Sulaymaniyah, 46012, Iraq; Department of Biology, College of Science, University of Sulaimani, Sulaymaniyah, Kurdistan Region, Iraq

**Keywords:** AMR, Antibiotic resistance, Infectious diseases, Pathogens, Public health

Dear Editor

Since the 1990s, Iraq has been grappling with a significant crisis that has had a detrimental impact on the lives of its citizens, affecting all strata of the community. The Iraqi education and healthcare systems face critical deficiencies due to unstable political conditions, security challenges, and an ongoing economic crisis. These stressful life events have significantly eroded the quality of the healthcare system, resulting in excess mortality across various conditions, including infectious diseases and the proliferation of nosocomial and hospital-acquired infections. Antibiotic resistance represents an additional apprehension of the abovementioned conditions, representing a critical dilemma in modern public health. Antibiotic resistance pertains to the development of antibiotic resistance in bacteria and potentially other microorganisms, rendering the efficacy of antibiotic therapy less impactful or even obsolete. There are already over 700,000 global fatalities attributed to antimicrobial resistance (AMR) annually, and this number is expected to rise to over 10 million by 2050 [1]. Throughout the past decades, there has been a substantial rise in antibiotic resistance, driven by the evolving mechanisms utilized by pathogens to resist antibiotics. This phenomenon is intricately linked to infectious diseases and virulence, especially in biofilm-producing bacteria [2]. The efficacy of many presently employed antibiotics is diminishing in the face of biofilm-associated and multidrug-resistant (MDR) microorganisms. Rampant antibiotic use in healthcare and agriculture has triggered a grave risk: surging antibiotic resistance [3]. In Kurdistan, antibiotics are readily available, leading to the proliferation of dangerously resistant bacteria [4]. This situation poses an imminent threat to public health and regional stability. Individuals often self-medicate with various antibiotics without understanding the potential risks and complications.

Pharmacologists anticipate a surge in antibiotic usage as winter arrives due to changing weather and a higher incidence of respiratory tract infections. Despite most infections being viral, antibiotics are often inappropriately prescribed, as they are commonly mistaken for bacterial infections. This trend is pronounced in cases of tonsillitis and the common cold, where non-prescription Amoxicillin dispensing is common [4]. Moreover, physicians responsible for prescribing antibiotics sometimes judiciously prescribe them without conducting essential diagnostic tests, like isolation and susceptibility tests, fuelling the quick development of antibiotic resistance in KRG. For instance, urinary tract infections (UTIs) in the KRG have revealed the presence of multidrug-resistant bacteria, including both Gram-positive and Gram-negative bacteria. The predominant species include *Escherichia coli*, *Acinetobacter baumannii, Staphylococcus* and *Enterococcus faecalis* [5].

On the other hand, the hospitals in the KRG capital city do not have effective programmes to manage antibiotics, resulting in poor-quality use and frequent inappropriate prescriptions, which only worsens the problem. Consequently, antibiotic efficacy diminishes, common infections can escalate into life-threatening conditions, and medical procedures that are currently routine, such as surgeries and cancer treatments, carry increased risks. This issue goes beyond regional concerns; it affects the broader global community. Addressing this impending crisis requires swift and comprehensive action from the relevant authorities and governmental bodies in the KRG. Strict regulations regarding the availability and distribution of antibiotics should be enforced, with a strong emphasis on their administration under the guidance of qualified healthcare professionals. Additionally, it is crucial to launch awareness campaigns that educate the public about the responsible use of antibiotics and the dangers of self-medication.

In conclusion, the escalating antibiotic resistance crisis in the KRG and Iraq at large poses a pressing and global concern. Swift and collaborative action is imperative to safeguard the efficacy of antibiotics and ensure a healthier future for the Kurdistan region and beyond. Recognised as an urgent global priority, addressing multidrug-resistant (MDR) infections demands immediate attention. Ongoing research promises to enhance our understanding of these challenges. Despite formidable obstacles, proactive measures are essential. By mobilising resources and fostering international cooperation, we can mitigate the dire consequences of antibiotic resistance, particularly concerning MDR microorganisms, and work toward a more resilient and healthier world.

## Ethics approval and consent to participle

Not applicable.

## Consent for publication

Not applicable.

## Funding

The authors did not receive any financial support for this work.

## CRediT authorship contribution statement

**Karzan Qurbani:** Writing – original draft, Conceptualization. **Seenaa Ali:** Writing – review & editing, Supervision. **Safin Hussein:** Writing – review & editing, Conceptualization. **Haider Hamzah:** Writing – review & editing, Supervision.

## Declaration of generative AI and AI-assisted technologies in the writing process

During the preparation of this work, the authors utilized ChatGPT to enhance the language and readability of certain paragraphs. Subsequently, the authors thoroughly reviewed and edited the content as necessary and take full responsibility for the publication's content.

## Declaration of competing interest

The authors declare that they have no known competing financial interests or personal relationships that could have appeared to influence the work reported in this paper.

## References

[1] Assar A., Abdelraoof M.I., Abdel-Maboud M., Shaker K.H., Menshawy A., Swelam A.H. (2020). Knowledge, attitudes, and practices of Egypt's future physicians towards antimicrobial resistance (KAP-AMR study): a multicenter cross-sectional study. Environ Sci Pollut Res.

[2] Gedefie A., Alemayehu E., Mohammed O., Bambo G.M., Kebede S.S., Kebede B. (2023). Prevalence of biofilm producing Acinetobacter baumannii clinical isolates: a systematic review and meta-analysis. PLoS One.

[3] Shao Y., Wang Y., Yuan Y., Xie Y. (2021). A systematic review on antibiotics misuse in livestock and aquaculture and regulation implications in China. Sci Total Environ.

[4] Almufty H., Hayhat L., Abdal W.G.A., Hussein D.S.H., Merza M.A.M. (2023). Magnitude of dispensing unprescribed antibiotics in community pharmacies in Duhok province; Kurdistan region of Iraq. J Contemp Med Sci.

[5] Al-Naqshbandi A.A., Chawsheen M.A., Abdulqader H.H. (2019). Prevalence and antimicrobial susceptibility of bacterial pathogens isolated from urine specimens received in rizgary hospital — erbil. J Infect Public Health.

